# Relation of plasma PCSK9 levels to lipoprotein subfractions in patients with stable coronary artery disease

**DOI:** 10.1186/1476-511X-13-188

**Published:** 2014-12-11

**Authors:** Rui-Xia Xu, Sha Li, Yan Zhang, Xiao-Lin Li, Yuan-Lin Guo, Cheng-Gang Zhu, Jian-Jun Li

**Affiliations:** Division of Dyslipidemia, State Key Laboratory of Cardiovascular Disease, Fu Wai Hospital, National Center for Cardiovascular Disease, Chinese Academy of Medical Sciences and Peking Union Medical College, Beijing, 100037 China

**Keywords:** Proprotein convertase subtilisin kexin type 9, LDL subfractions, HDL subfractions, Coronary artery disease

## Abstract

**Background:**

Plasma PCSK9 levels was positively associated with low-density lipoprotein (LDL) cholesterol (LDL-C) and atherosclerosis, while PCSK9 may also be implicated in the metabolism of lipoprotein subfractions. The study was to examine the association of plasma PCSK9 with lipoprotein subfractions in patients with stable coronary artery disease (CAD).

**Methods:**

A total of 281 consecutive, stable CAD patients who were not treated with lipid-lowering drugs were enrolled. The baseline clinical characteristics were collected, the plasma PCSK9 levels were determined using ELISA, and the LDL and high-density lipoprotein (HDL) subfractions were analyzed by Lipoprint System. The association of plasma PCSK9 levels with the lipoprotein subfractions was investigated.

**Results:**

In the overall population, plasma PCSK9 levels were positively associated with the concentration of LDL-C, intermediate LDL-C, small LDL-C, and LDL score, while negatively correlated with mean LDL particle size. PCSK9 levels were positively associated with the concentration of HDL-C, intermediate HDL-C and small HDL-C. Multivariable regression analysis revealed that the plasma PCSK9 levels were significantly and independently associated with the concentration of intermediate LDL-C (β = 0.152, p = 0.013), small LDL-C (β = 0.179, p = 0.004), LDL score (β = 0.121, p = 0.043), and mean LDL particle size (β = -0.130, p = 0.035), while not HDL subfractions. Interestingly, when investigated in male and female patients separately, these relationships were only found in male but not in female, and the small HDL-C exhibited an association with PCSK9 levels in male patients (β = 0.149, p = 0.045).

**Conclusions:**

PCSK9 levels were independently associated with the changes of lipoprotein subfractions, suggesting a potential interaction between PCSK9 and lipoprotein subfractions in CAD.

## Introduction

Coronary artery disease (CAD) is one of the leading causes of death worldwide. It is well known that hypercholesterolemia is a major pathogenic risk factor for CAD, and it increases the incidence of myocardial infarction and death [1]. Proprotein convertase subtilisin kexin type 9 (PSCK9), originally discovered as a third gene involved in autosomal dominant hypercholesterolemia [2], has gained considerable attention over the past decade. PSCK9 is a liver-derived secreted protease which has been identified as a key regulator of low density lipoprotein (LDL) receptor (LDLR) processing [3]. PCSK9 binds directly to the epidermal growth factor repeat A of the LDLR, subsequently promoting its degradation [4–7]. This process reduces the number of LDLR, the major receptor mediating the clearance of LDL-cholesterol (LDL-C), and results in increased LDL-C levels in the circulation [8, 9]. In agreement with the importance of the PCSK9 for LDL-C metabolism, a positive relationship of plasma total cholesterol (TC), LDL-C and apolipoprotein B (apoB) levels with plasma PCSK9 has been repeatedly demonstrated [10].

LDL or high density lipoprotein (HDL) is a heterogeneous particle of lipoprotein populations with respect to size, density and chemical composition [11]. Many studies have shown that the smaller LDL particles are more atherogenic than the larger ones, and a higher LDL particle number was consistently associated with increased risk for cardiovascular disease independent of other lipid measurements [12, 13]. Moreover, a growing body of evidence from epidemiological data, animal studies, and clinical trials supports HDL as the next target to reduce residual cardiovascular risk in statin-treated, high-risk patients. However, little is known about the relationship of plasma PSCK9 and LDL or HDL particles. The present study was, therefore, to investigate the potential relationship between plasma PCSK9 levels and LDL and HDL subfractions in patients with stable CAD without statins therapy.

## Methods

### Ethical approval

The study complied with the Declaration of Helsinki and was approved by the Ethics Committee of Fu Wai Hospital and Cardiovascular Institute, Beijing, China. Informed written consent was obtained from all patients enrolled in this study.

### Study design and population

From October 2012 through February 2014, we consecutively recruited 281 patients with significant CAD, which was defined as one or more diseased epicardial vessels with a diameter of more than 2 mm that had at least a 50% diameter stenosis by elective coronary angiography due to angina-like chest pain and/or positive treadmill exercise test or clinically indicated coronary computed tomography (CT) angiography. Patients with acute coronary syndrome (ACS), heart failure (The left ventricular ejection fraction, LVEF <45%), infectious or systematic inflammatory disease, thyroid dysfunction, severe liver and/or renal insufficiency and malignant disease were excluded from the current study. Inclusion criteria of patients were as follows: 1) with definite clinical evidence of atherosclerotic lesions reached to the diagnosis criteria of CAD; 2) without treatment history of statins and/or other lipid-lowering drugs at least 3 months prior to entering the study; 3) with assessment for clinical history, anthropometric characteristics and standard cardiovascular risk factors. The definition of hypertension was repeated blood pressure measurements ≥ 140/90 mm Hg or currently taking anti- hypertensive drugs. Diabetes was defined as fasting plasma glucose ≥ 7.0 mmol/ L in multiple determinations or patients were receiving an active treatment with insulin or oral hypoglycemic agents. Dyslipidemia was defined as the presence of fasting TC ≥ 200 mg/dl and/or triglyceride (TG) ≥ 150 mg/dl.

### Laboratory examinations

Blood samples were obtained in all patients from cubital vein after a 12-hour overnight fasting and collected into EDTA-containing tubes. All samples were subsequently stored at -80°C and analyzed immediately after thawing. Concentrations of plasma TC, TG, HDL cholesterol (HDL-C), LDL-C, apolipoprotein A-I (apoA-I), apolipoprotein B (apoB), lipoprotein (a) [Lp (a)], and free fatty acid (FFA) were measured using an automatic biochemistry analyzer (Hitachi 7150, Tokyo, Japan). Of which, TC, TG, HDL-C, LDL-C, and FFA levels were measured by enzymatic assay. ApoA-I, apoB, and Lp (a) levels were measured by turbidimetric immunoassay. Plasma PCSK9 concentrations were measured using a high sensitivity, quantitative sandwich enzyme-linked immunosorbent assay (CircuLex ELISA, CycLex Co., Nagano, Japan). The mean minimum detectable dose of PCSK9 was 0.154 ng/ml.

### LDL subfraction analysis

Blood samples were also used for subfraction analysis. LDL subfraction analysis was performed electrophoretically by the use of high-resolution 3% polyacrylamide gel tubes and the Lipoprint LDL System (Quantimetrix Corporation, Redondo Beach, CA, USA) according to the manufacturer’s instructions as previously described [14, 15]. This method was based on electrophoresis of a liquid loading gel with lipophilic dye in the precast linear polyacrylamide gel (stacking gel and separating gel). A typical Lipoprint profile of decreasing size and increasing density with 1 very low density lipoprotein (VLDL) band, 3 Midbands, up to 7 LDL bands, and 1 HDL band was obtained. The various stained bands (lipoprotein subfractions) presented in the sample were indentified by their electrophoretic mobility (Rf) using VLDL as the starting reference point (VLDL = 0) and HDL as the leading reference point (HDL = 1). The relative area for each lipoprotein subfraction was determined and multiplied by TC concentration of the sample to yield the amount of cholesterol for each band in mg/dl. As a result, the cholesterol mass of each LDL subfraction and the mean LDL particle size (Å) were calculated on the basis of the different areas under the curve with different Rf. Subfraction 1 represented large LDL particles, subfraction 2 indicated intermediate LDL particles, and subfraction 3-7 meant small LDL particles. The proportion of sd-LDL particles (subfractions 3–7) to the whole LDL area (subfractions 1–7) was also calculated in our sample (LDL score).

### HDL subfraction analysis

Similar to LDL subfraction analysis, the cholesterol contents of HDL subfractions were also determined electrophoretically by the use of high-resolution 3% polyacrylamide gel tubes and the Lipoprint HDL System (Quantimetrix Corporation, Redondo Beach, CA, USA) as previously described [14]. After the electrophoresis was completed, the various stained HDL subfractions (bands) presented presented in the sample were indentified by Rf using LDL/VLDL as the starting reference point (LDL/VLDL = 0) and Albumin as the leading reference point (Albumin = 1). The relative area for each HDL subfraction was determined and multiplied by HDL-C concentration of the sample to yield the amount of cholesterol for each band in mg/dl. Using this assay, HDL was divided into 10 subfractions. Subfraction 1-3 represented large HDL particles, subfraction 4-7 indicated intermediate HDL particles, and subfraction 8-10 mean small HDL particles.

### Statistical analysis

The data are expressed as mean ± standard deviation or median (Q1-Q3 quartiles) for continuous variables and number (percentage) for categorical variables. The Mann-Whitney *U* test was used for the comparison of clinical parameters between two groups. The categorical variables were compared using the chi-square test. A linear univariable regression analysis was performed to determine the relationship between PCSK9 levels and lipoprotein subfractions. Multivariable regression analysis was used to assess the independent contribution of the variables. A p-value < 0.05 was considered statistically significant. Statistical analysis was performed with SPSS version 19.0 software (SPSS Inc., Chicago, IL, USA).

## Results

### Baseline characteristics

We enrolled 281 patients with stable CAD in this study. Their clinical characteristics, plasma PCSK9 levels, (apo)lipoproteins and lipoprotein subfractions are shown in Table 1. The mean age of the study population was 57.97 ± 9.55 years, and 70.8% of the patients were men. The plasma PCSK9 levels ranged from 99.23 to 477.70 ng/ml, and the distribution of PCSK9 levels in this population was right-skewed (median: 215.53 ng/ml).

**Table 1:** **Baseline characteristics of the study population**

Characteristics	Patients (n = 281)
**Demographics**	
Age (years)	57.97 ± 9.55
Men [n (%)]	199 (70.8)
BMI (kg/m^2^)	25.96 ± 3.42
**Coronary risk factors**	
Hypertension [n (%)]	190 (67.6)
Diabetes [n (%)]	75 (26.7)
Dyslipidemia [n (%)]	197 (70.1)
Smoking [n (%)]	139 (49.5)
Family history of CAD [n (%)]	52 (18.5)
**Laboratory parameters**	
TC (mg/dl)	190.23 ± 43.09
TG (mg/dl)	147.79 (104.87-207.96)
Non-HDL-C (mg/dl)	147.77 ± 41.85
Apo A-I (g/L)	1.39 ± 0.26
Apo B (g/L)	1.00 ± 0.28
LDL-C (mg/dl)	122.91 ± 38.87
Large LDL-C (mg/dl)	27.76 ± 9.66
Intermediate LDL-C (mg/dl)	20.81 ± 9.26
Small LDL-C (mg/dl)	9.29 ± 9.97
LDL score (% of sd-LDL)	14.48 ± 13.56
Mean LDL size (Å)	266.06 ± 6.10
HDL-C (mg/dl)	42.46 ± 14.68
Large HDL-C (mg/dl)	13.70 ± 7.50
Intermediate HDL-C (mg/dl)	20.90 ± 7.35
Small HDL-C (mg/dl)	8.17 ± 3.49
ALT (U/L)	24.92 ± 17.34
AST (U/L)	18.54 ± 8.72
ALP (IU/L)	64.67 ± 17.78
GGT (IU/L)	33.56 ± 23.96
Cr (μmol/L)	72.38 ± 16.63
BUN (mmol/L)	5.94 ± 1.47
PCSK9 (ng/ml)	215.53 (180.14-260.39)

The data shown are the mean ± SD, median (Q1-Q3 quartiles) or n (%).

BMI: body mass index. CAD: coronary artery disease. TC: total cholesterol. TG: triglycerides. Non-HDL-C: non high-density lipoprotein cholesterol. ApoA-I: apolipoprotein A1. ApoB: apolipoprotein B. LDL-C: low-density lipoprotein-cholesterol. HDL-C: high-density lipoprotein cholesterol. ALT: alanine aminotransferase. AST: aspartate aminotransferase. ALP: alkaline phosphatase. GGT: glutamyl transferase. Cr: creatinine. BUN: blood urea nitrogen. PSCK9: proprotein convertase subtilisin kexin type 9.

### Correlations of plasma PCSK9 levels with lipid profile and lipoprotein subfractions

For the Spearman correlation analysis, plasma PCSK9 was not only correlated positively with TC, LDL-C, apo B, non-HDL-C, but also with HDL-C and apo A-I (Table 2) in overall population. However, PCSK9 was unrelated to TG. Furthermore, PCSK9 was positively correlated with intermediate, small LDL-C, and LDL score (intermediate LDL-C r = 0.145, p = 0.015; small LDL-C r = 0.166, p = 0.005; LDL score r = 0.120, p = 0.046), while not with large LDL-C and mean LDL particle size (large LDL-C r = 0.089, p = 0.140; mean LDL particle size r = -0.104, p = 0.084). A positive relationship between PCSK9 and intermediate and small HDL-C was also observed in our study (r = 0.157, p = 0.013; r = 0.124, p = 0.048; respectively). However, there were no significant associations between the PCSK9 levels and age, body mass index (BMI), blood pressure, and plasma glucose levels.

**Table 2:** **Univariable correlations of PCSK9 with lipid profile and lipoprotein subfractions in patients with CAD**

Variables	r	P-value
Age (years)	0.006	0.924
BMI (kg/m^2^)	-0.064	0.287
SBP (mm Hg)	-0.080	0.180
DBP (mm Hg)	-0.034	0.568
Glucose (mmol/L)	0.047	0.434
TC (mg/dl)	0.249	**0.000**
TG (mg/dl)	0.075	0.213
Non-HDL-C (mg/dl)	0.221	**0.000**
Apo A-I (g/L)	0.184	**0.003**
Apo B (g/L)	0.260	**0.000**
LDL-C (mg/dl)	0.219	**0.000**
Large LDL-C (mg/dl)	0.089	0.140
Intermediate LDL-C (mg/dl)	0.145	**0.015**
Small LDL-C (mg/dl)	0.166	**0.005**
LDL score (% of sd-LDL)	0.120	**0.046**
Mean LDL size (Å)	-0.104	0.084
HDL-C (mg/dl)	0.149	**0.012**
Large HDL-C (mg/dl)	0.045	0.476
Intermediate HDL-C (mg/dl)	0.157	**0.013**
Small HDL-C (mg/dl)	0.124	**0.048**

Spearman correlation analysis is shown. The bold values indicate statistical significance and are bolded to improve the readability of the table.

BMI: body mass index. SBP: systolic blood pressure. DBP: diastolic blood pressure. TC: total cholesterol. TG: triglycerides. Non-HDL-C: non high-density lipoprotein cholesterol. ApoA-I: apolipoprotein A1. ApoB: apolipoprotein B. LDL-C: low-density lipoprotein-cholesterol. HDL-C: high-density lipoprotein cholesterol.

To determine the strengthen of the relationship of PCSK9 levels with lipids and lipoprotein subfractions in patients with stable CAD, we performed a multivariable linear regression analysis (Table 3). After adjustment for age, gender, BMI, hypertension, diabetes mellitus and positive family history, PCSK9 was significantly related with TC (β = 0.215, p < 0.001), LDL-C (β = 0.197, p = 0.001), apo B (β = 0.245, p < 0.001), apo A-I (β = 0.137, p = 0.025), intermediate LDL-C (β = 0.152, p = 0.013), small LDL-C (β = 0.179, p = 0.004), and LDL score (β = 0.121, p = 0.043). Interestingly, a negative association between PCSK9 and mean LDL size arose (β = -0.130, p = 0.035) after adjustment for above confounders. However, the relationship between PCSK9 and each HDL subtraction vanished (Table 3) after the adjustment.

**Table 3:** **Independent associations of the PCSK9 levels with the lipid profile and lipoprotein subfractions in patients with CAD**

Variables	Multivariable linear regression analysis	
	Coefficients	P-value
TC (mg/dl)	0.215	**0.000**
TG (mg/dl)	0.042	0.494
Non-HDL-C (mg/dl)	0.202	**0.001**
Apo A-I (g/L)	0.137	**0.025**
Apo B (g/L)	0.245	**0.000**
LDL-C (mg/dl)	0.197	**0.001**
Large LDL-C (mg/dl)	0.033	0.590
Intermediate LDL-C (mg/dl)	0.152	**0.013**
Small LDL-C (mg/dl)	0.179	**0.004**
LDL score (% of sd-LDL)	0.121	**0.043**
Mean LDL size (Å)	-0.130	**0.035**
HDL-C (mg/dl)	0.056	0.334
Large HDL-C (mg/dl)	-0.050	0.380
Intermediate HDL-C (mg/dl)	0.069	0.276
Small HDL-C (mg/dl)	0.062	0.332

The multivariable linear regression analysis was adjusted for age, gender, BMI, hypertension, diabetes mellitus, current smoking and positive family history. The lipids and lipoprotein subfractions were the dependent variables.

TC: total cholesterol. TG: triglycerides. Non-HDL-C: non high-density lipoprotein cholesterol. ApoA-I: apolipoprotein A1. ApoB: apolipoprotein B. LDL-C: low-density lipoprotein-cholesterol. HDL-C: high-density lipoprotein cholesterol.

### Gender analysis in the correlations of plasma PCSK9 with lipid profile and lipoprotein subfractions

A gender comparison of the relationship between the PCSK9 and lipids and lipoprotein subfractions was also performed in the present study due to the reported gender differences in prevalence of CAD and levels of PCSK9. As presented in Table 4, women with stable CAD had not only an older age of onset and more significant disturbances in their lipid profiles, but also higher PCSK9 levels compared to men.

**Table 4:** **Comparisons of the clinical characteristics between male and female patients with CAD**

Variables	Male (n = 199)	Female (n = 82)	P-value
Age (years)	56.79 ± 9.32	60.84 ± 9.56	**0.001**
BMI (kg/m^2^)	26.20 ± 3.14	25.35 ± 3.99	0.088
Hypertension [n (%)]	135 (67.8)	55 (67.1)	0.890
Diabetes [n (%)]	53 (26.6)	22 (26.8)	0.973
Dyslipidemia [n (%)]	135 (67.8)	62 (75.6)	0.251
Smoking [n (%)]	134 (67.3)	5 (6.1)	**0.000**
Family history of CHD [n (%)]	30 (15.1)	22 (26.8)	**0.028**
TC (mg/dl)	183.73 ± 40.76	205.99 ± 44.72	**0.000**
TG (mg/dl)	147.79 (106.19-208.85)	146.02 (98.23-207.30)	0.428
Non-HDL-C (mg/dl)	144.24 ± 40.11	156.33 ± 44.92	**0.027**
Apo A-I (g/L)	1.34 ± 0.21	1.52 ± 0.31	**0.000**
Apo B (g/L)	0.98 ± 0.27	1.07 ± 0.30	**0.019**
LDL-C (mg/dl)	110.07 ± 37.09	134.65 ± 40.78	**0.001**
Large LDL-C (mg/dl)	26.80 ± 9.35	30.10 ± 10.05	**0.009**
Intermediate LDL-C (mg/dl)	20.58 ± 9.01	21.38 ± 9.87	0.512
Small LDL-C (mg/dl)	9.49 ± 10.57	8.79 ± 8.37	0.593
LDL score (% of sd-LDL)	14.92 ± 14.23	13.42 ± 11.75	0.366
Mean LDL size (Å)	265.74 ± 6.44	266.85 ± 5.15	0.168
HDL-C (mg/dl)	39.49 ± 11.68	49.67 ± 18.37	**0.000**
Large HDL-C (mg/dl)	12.10 ± 5.40	17.49 ± 10.05	**0.000**
Intermediate HDL-C (mg/dl)	19.76 ± 6.27	23.60 ± 8.91	**0.001**
Small HDL-C (mg/dl)	7.99 ± 3.57	8.59 ± 3.28	0.214
PCSK9 (ng/ml)	205.21 (174.32-247.02)	236.93 (193.10-283.64)	**0.000**

BMI: body mass index. TC: total cholesterol. TG: triglycerides. Non-HDL-C: non high-density lipoprotein cholesterol. ApoA-I: apolipoprotein A1. ApoB: apolipoprotein B. LDL-C: low-density lipoprotein-cholesterol. HDL-C: high-density lipoprotein cholesterol.

**Figure 1:**
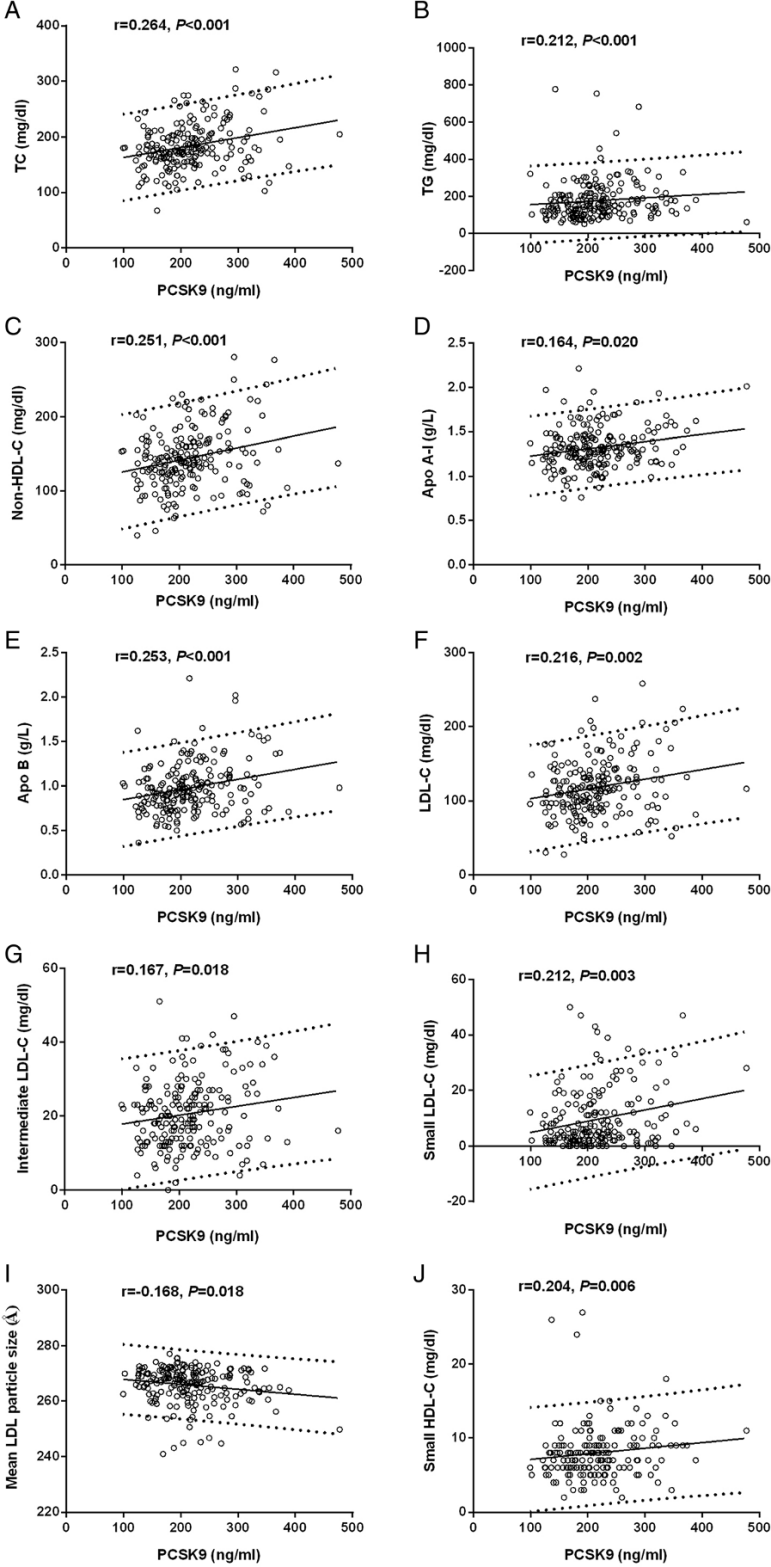
**Correlations of the plasma PCSK9 levels with lipid profile and lipoprotein subfractions in male patients with CAD.** Spearman correlation analysis was shown for the association of PCSK9 with TC **(A)**, TG **(B)**, non-HDL-C **(C)**, Apo A-I **(D)**, Apo B **(E)**, LDL-C **(F)**, intermediate LDL-C **(G)**, small LDL-C **(H)**, mean LDL particle size **(I)** and small HDL-C **(J)**.

In the present study, we found no significant correlation of the PCSK9 levels with the cholesterol concentration of LDL and HDL subfractions in women, even with bivariable correlation analysis (data not shown). However, in men, there were positive relationships between the PCSK9 levels and the concentrations of TC (β = 0.264, p < 0.001), TG (β = 0.212, p < 0.001), non-HDL-C (β = 0.251, p < 0.001), apo A-I (β = 0.164, p = 0.020), apo B (β = 0.253, p < 0.001), LDL-C (β = 0.216, p = 0.002), intermediate LDL-C (β = 0.167, p = 0.018), small LDL-C (β = 0.212, p = 0.003) and mean LDL particle size (β = -0.168, p = 0.018), as well as small HDL-C (β = 0.204, p = 0.006) (Figure 1). Furthermore, after adjusting for the traditional risk factors such as age, BMI, hypertension, diabetes mellitus, current smoking and positive family history, there remained significant correlations of the PCSK9 levels with TC (β = 0.289, p < 0.001), TG (β = 0.148, p = 0.031), non-HDL-C (β = 0.264, p < 0.001), apo A-I (β = 0.223, p = 0.001), apo B (β = 0.269, p < 0.001), LDL-C (β = 0.230, p = 0.001), intermediate LDL-C (β = 0.183, p = 0.009), small LDL-C (β = 0.240, p = 0.001), LDL score (β = 0.177, p = 0.015), mean LDL particle size (β = -0.174, p = 0.015) and small HDL-C (β = 0.149, p = 0.045) (Table 5).

**Table 5:** **Independent associations of the PCSK9 levels with the lipid profile and lipoprotein subfractions across gender**

Variables	Multivariable linear regression analysis			
	Male		Female	
	Coefficients	P-value	Coefficients	P-value
TC (mg/dl)	0.289	**0.000**	0.042	0.733
TG (mg/dl)	0.148	**0.031**	-0.160	0.183
Non-HDL-C (mg/dl)	0.264	**0.000**	0.033	0.787
Apo A-I (g/L)	0.223	**0.001**	0.050	0.683
Apo B (g/L)	0.269	**0.000**	0.146	0.233
LDL-C (mg/dl)	0.230	**0.001**	0.065	0.587
Large LDL-C (mg/dl)	-0.006	0.929	0.140	0.248
Intermediate LDL-C (mg/dl)	0.183	**0.009**	0.041	0.739
Small LDL-C (mg/dl)	0.240	**0.001**	-0.063	0.605
LDL score (% of sd-LDL)	0.177	**0.015**	-0.060	0.603
Mean LDL size (Å)	-0.174	**0.015**	0.030	0.806
HDL-C (mg/dl)	0.100	0.157	0.021	0.862
Large HDL-C (mg/dl)	-0.044	0.550	-0.074	0.502
Intermediate HDL-C (mg/dl)	0.087	0.253	0.043	0.730
Small HDL-C (mg/dl)	0.149	**0.045**	-0.172	0.166

The multivariable linear regression analysis was adjusted for age, BMI, hypertension, diabetes mellitus, current smoking and positive family history. The lipids and lipoprotein subfractions are the dependent variables.

TC: total cholesterol. TG: triglycerides. Non-HDL-C: non high-density lipoprotein cholesterol. ApoA-I: apolipoprotein A1. ApoB: apolipoprotein B. LDL-C: low-density lipoprotein-cholesterol. HDL-C: high-density lipoprotein cholesterol.

## Discussion

The major finding of the present study was that the plasma PCSK9 levels were positively and independently associated with intermediate and small LDL subfractions but not large LDL subfraction in patients with stable CAD. Interestingly, our study firstly found these associations were significantly only in men but not in women, suggesting the interacting impact of PCSK9 with lipoprotein subfractions may be a novel mechanism for gender disparity in the development of CAD.

PCSK9 is an enzyme with an important role in lipoprotein metabolism [16]. In human, gain-of-function mutations in the PCSK9 gene caused a high LDL-C level and a form of familial hypercholesterolemia [17], whereas loss-of-function variants lead to a low LDL-C level and a reduced incidence of CAD [18, 19]. The positive associations of the plasma PCSK9 levels with TC and LDL-C were previously observed in patients with familial hypercholesterolemia and CAD [20, 21], even in the healthy subjects [22, 23]. Recently, studies have shown that circulating PCSK9 levels were significantly correlated with TC, LDL-C and non-HDL-C [10, 24, 25]. As anticipated, in the present study, we observed strong positive correlations of plasma PSCK9 with TC, non-HDL-C, LDL-C, apo B levels. We also found that PCSK9 was not correlated positively with TG levels, which was in disagreement with previous data [25, 26]. Mayne et al showed that plasma PCSK9 levels were positively correlated with TC and LDL-C in male normolipidemic subjects but not in female [27]. Similarly, our study also demonstrated that PCSK9 was significantly associated with TC, LDL-C and non-HDL-C in male patients with CAD while not in female, suggesting that a gender difference in PCSK9 regulation and function with PCSK9 correlated to TC and LDLC in men but not women. The explanation for these gender differences is unknown. Previous studies demonstrated that estrogen augmented LDL receptor levels, while androgens attenuated these effects [28, 29], although the effect(s) of these hormones on *PCSK9* transcription and/or translation have not been studied.

It has been demonstrated that the changes of lipoprotein subfractions are strongly related the increased risk of CAD [30, 31]. The major novel findings are that plasma PCSK9 levels were significantly and independently associated with the cholesterol concentrations of intermediate and small LDL subfractions as well as mean LDL particle size in patients with stable CAD. Actually, circulating LDL as well as HDL consists of a heterogeneous group of particles with respect to size, density, and chemical composition [32]. The differences of LDL particles lead to the recognition of two distinct phenotypes: phenotype A, associated with large, buoyant LDL particles (lb-LDL); and phenotype B, in which small and dense LDL particles (sd-LDL) predominate [33]. Presently, the small LDL particles have been recognized more atherogenic than large ones and can predict atherosclerosis progression and incident CHD [33, 34]. Recently, Kwakernaak et al found that plasma PCSK9 was significantly correlated with LDL particle concentrations, and was independently related to intermediate density lipoprotein (IDL) by nuclear magnetic resonance spectroscopy analysis in healthy subjects [25]. A new study demonstrated that men with higher PCSK9 levels did not have higher cholesterol in large or medium size LDL particles, but had higher cholesterol levels in small LDL particles, indicating that plasma PCSK9 levels were modestly associated with LDL particle size in abdominally obese, dyslipidemic men [35]. Apparently, our results confirmed and extended previous study, suggesting that PCSK9 predominantly related to the metabolism of intermediate and small LDL particles in patients with CAD.

The associations of plasma PCSK9 with HDL subfractions are not well characterized, although the emerging opinion is that the quality of HDL particles may be more important than the quantity of HDL for the development of CAD. Several recent studies in mice and non-human primates have shown that PCSK9 is involved in HDL metabolism. B6 male mice fed a high fat diet and then treated with a Pcsk9 antisense oligonucleotide inhibitor for 6 weeks showed a 54% reduction in HDL cholesterol concentration [36]. In male cynomolgus macaques, treatment with neutralizing antibodies against PCSK9 reduced HDL cholesterol concentrations for the first seven days of treatment [37]. To our knowledge, our data firstly found that plasma PCSK9 levels were positively associated with HDL-C in human study. In male CAD patients not female, PCSK9 was positively correlated with the concentration of small HDL-C. The mechanism underlying this observation was not completely understood.

Numerous studies have now suggested a remark gender difference concerning the clinical characteristics of CAD [38–40]. Women with CAD were more likely to have cardiovascular risk factors but a milder atheroma burden than men [39]. Women developed CAD when they are about ten years older than men and, typically, after menopause [41]. Though the risk of CAD is greater in men than that in women, the exact mechanisms are not fully understood. LDL becomes more atherogenic and toxic as its particle size decreases and its electronegativity increases [42]. A recent study found that LDL electronegativity is higher in male patients with metabolic syndrome (MetS) than female patients with MetS, which underlie the increased propensity to CAD observed in male patients [43]. Previous study showed that increased sdLDL fraction and decreased LDL particle size were positively correlated with the extent and severity of CAD [30]. Pitavastatin improved LDL subfraction profiles, and this in turn may reduced the cardiovascular risk in patients with type 2 diabetes and dyslipidemia [44]. PCSK9 is an important regulator of LDL-C concentrations in plasma, which was found significantly different between male and female [27, 45]. Therefore, we further examine the gender disparities for the relationships between PCSK9 and lipoprotein subfractions. Interestingly, our data firstly found that PCSK9 was positively and independently associated with intermediate LDL-C, small LDL-C, and small HDL-C, while negatively with LDL particle size in male patients with CAD, but not in female. This finding may have important clinical implication in spite of unknown exact mechanisms. Whether it is a potential cause for explanation the gender differences in the development of CAD appear attractive for further study.

There were several limitations of the present study. First, it is a cross-sectional study of patients referred for coronary angiography. Second, the relative small sample size from a single center may limit the ability to detect weak correlations in both univariable and multivariable analysis. Finally, the study population represents an inhomogeneous cohort with regard to gender. Therefore, the results of our study should careful explanation and needed to be further investigation.

Summarily, in the present study, plasma PCSK9 level was independently positively associated with the cholesterol concentration of intermediate and small LDL subfractions and LDL particle size in patients with stable CAD. An analysis across gender showed that this relationship was only found in male patients but not in female, suggesting the interacting impact of PCSK9 with lipoprotein subfractions may be a novel mechanism for gender disparity in the development of CAD. Whether this impact has a causal effects may be needed to further evaluate.
